# 4-Methyl-2-oxo-2*H*-chromen-7-yl 4-fluoro­benzene­sulfonate

**DOI:** 10.1107/S1600536812004394

**Published:** 2012-02-10

**Authors:** Suman Sinha, Hasnah Osman, Habibah A. Wahab, Madhukar Hemamalini, Hoong-Kun Fun

**Affiliations:** a School of Pharmaceutical Sciences, Universiti Sains Malaysia, 11800 USM, Penang, Malaysia; b School of Chemical Sciences, Universiti Sains Malaysia, 11800 USM, Penang, Malaysia; c X-ray Crystallography Unit, School of Physics, Universiti Sains Malaysia, 11800 USM, Penang, Malaysia

## Abstract

In the asymmetric unit of the title compound, C_16_H_11_FO_5_S, the 2*H*-chromene ring is essentially planar, with a maximum deviation of 0.040 (2) Å. The dihedral angle between the 2*H*-chromene ring and the 4-fluoro­phenyl ring is 2.17 (8)°. One of the sulfonamide O atoms is approximately coplanar with the benzene ring [C—C—S—O torsion angle = 166.00 (14)°], whereas the other O atom lies well below the plane [C—C—S—O = −61.35 (17)°]. In the crystal, mol­ecules are connected by weak C—H⋯O hydrogen bonds, forming two-dimensional networks parallel to the *ac* plane.

## Related literature

For details and applications of coumarines, see: Gu *et al.* (2007 ▶); Wrobel *et al.* (2002 ▶); Kostova (2005 ▶). For related structures, see: Sinha *et al.* (2011**a* ▶,b* ▶); Al-Najjar *et al.* (2012 ▶). For the synthetic procedure, see: Sinha *et al.* (2011**a* ▶,b* ▶); Fusegi *et al.* (2009 ▶). For the stability of the temperature controller used in the data collection, see: Cosier & Glazer (1986 ▶).
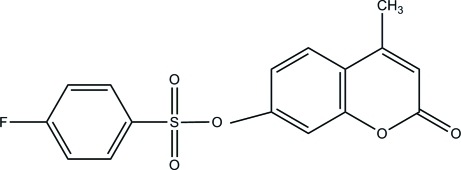


## Experimental

### 

#### Crystal data

C_16_H_11_FO_5_S
*M* *_r_* = 334.31
Monoclinic, 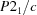

*a* = 17.2983 (4) Å
*b* = 5.3397 (1) Å
*c* = 17.1669 (4) Å
β = 118.195 (1)°
*V* = 1397.52 (5) Å^3^
*Z* = 4
Mo *K*α radiation
μ = 0.27 mm^−1^
*T* = 100 K
0.36 × 0.19 × 0.16 mm

#### Data collection

Bruker SMART APEXII CCD area-detector diffractometer
Absorption correction: multi-scan (*SADABS*; Bruker, 2009 ▶) *T*_min_ = 0.911, *T*_max_ = 0.959
26795 measured reflections
4303 independent reflections
3494 reflections with *I* > 2σ(*I*)
*R*_int_ = 0.047

#### Refinement

*R*[*F* ^2^ > 2σ(*F* ^2^)] = 0.056
*wR*(*F* ^2^) = 0.156
*S* = 1.04
4303 reflections
209 parameters
H-atom parameters constrained
Δρ_max_ = 1.14 e Å^−3^
Δρ_min_ = −0.72 e Å^−3^

### 

Data collection: *APEX2* (Bruker, 2009 ▶); cell refinement: *SAINT* (Bruker, 2009 ▶); data reduction: *SAINT*; program(s) used to solve structure: *SHELXTL* (Sheldrick, 2008 ▶); program(s) used to refine structure: *SHELXTL*; molecular graphics: *SHELXTL*; software used to prepare material for publication: *SHELXTL* and *PLATON* (Spek, 2009 ▶).

## Supplementary Material

Crystal structure: contains datablock(s) global, I, a. DOI: 10.1107/S1600536812004394/rz2704sup1.cif

Structure factors: contains datablock(s) I. DOI: 10.1107/S1600536812004394/rz2704Isup2.hkl

Supplementary material file. DOI: 10.1107/S1600536812004394/rz2704Isup3.cml

Additional supplementary materials:  crystallographic information; 3D view; checkCIF report

## Figures and Tables

**Table 1:** Hydrogen-bond geometry (Å, °)

*D*—H⋯*A*	*D*—H	H⋯*A*	*D*⋯*A*	*D*—H⋯*A*
C1—H1*A*⋯O3^i^	0.95	2.48	3.314 (2)	147
C4—H4*A*⋯O5^ii^	0.95	2.57	3.214 (3)	126
C8—H8*A*⋯O4^ii^	0.95	2.37	3.288 (3)	162
C11—H11*A*⋯O5^iii^	0.95	2.45	3.349 (3)	158
C15—H15*A*⋯O3^iv^	0.95	2.59	3.502 (3)	160
C16—H16*A*⋯O5^iii^	0.98	2.60	3.522 (3)	157

Symmetry codes: (i) 
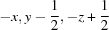
; (ii) 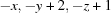; (iii) 
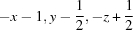
; (iv) 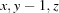.

**Fig. 1.**
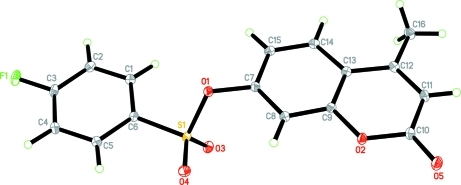
The asymmetric unit of the title compound, showing 30% probability displacement ellipsoids.

**Fig. 2.**
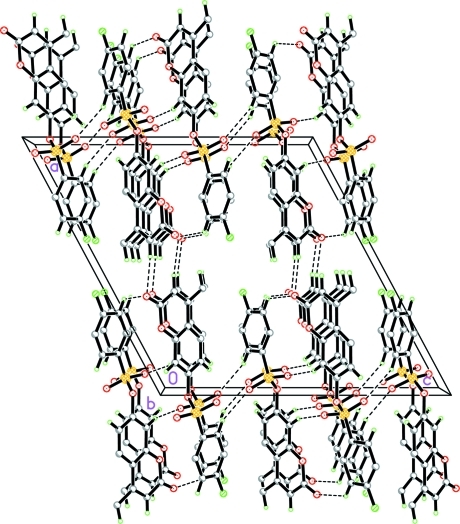
The crystal packing of the title compound. Intermolecular hydrogen bonds are shown as dashed lines.

## supplementary crystallographic information

### Comment

This work is to further explore the structural features of sulphur- containing small molecule derivatives which are being recently published from our laboratory (Sinha *et al.*, 2011**a*,b*; Al-Najjar *et al.*, 2012). Recently, the O–SO_2_ group have attracted attention in organic chemistry (Gu *et al.*, 2007) and medicinal chemistry (Wrobel *et al.*, 2002). Coumarines are also proven to be cytotoxic agents (Kostova, 2005). In this paper, we report the crystal structure of the title compound, which belongs to this class of compounds.

The asymmetric unit of the title compound is shown in Fig. 1. The 2*H*-chromene (O2/C7–C15) ring is essentially planar, with a maximum deviation of 0.040 (2) Å for atom C12. The dihedral angle between the 2*H*-chromene (O2/C7–C15) ring and fluoro-substituted phenyl (C1–C6) ring is 2.17 (8)°. The S atom adopts a distorted tetrahedral geometry. The sulfonamide O4 atom is approximately co-planar with the benzene ring [the O4-S1-C1-C6 torsion angle is 166.00 (14)°] whereas the O3 atom lies well below the plane [O3-S1-C1-C6 = -61.35 (17)°].

In the crystal, (Fig. 2), the molecules are connected *via* weak intermolecular C—H···O hydrogen bonds (Table 1) to form two-dimensional networks parallel to the *ac* plane.

### Experimental

Detailed synthetic procedure has been described in Sinha *et al.* (2011**a*,b*) and Fusegi *et al.* (2009).

### Refinement

All hydrogen atoms were positioned geometrically [C–H = 0.95 or 0.98 Å] and were refined using a riding model, with *U*_iso_(H) = 1.2 or 1.5 *U*_eq_(C). A rotating group model was applied to the methyl groups.

### Crystal data

**Table:**

C_16_H_11_FO_5_S	*F*(000) = 688
*M**_r_* = 334.31	*D*_x_ = 1.589 Mg m^−^^3^
Monoclinic, *P*2_1_/*c*	Mo *K*α radiation, λ = 0.71073 Å
Hall symbol: -P 2ybc	Cell parameters from 9952 reflections
*a* = 17.2983 (4) Å	θ = 2.4–30.6°
*b* = 5.3397 (1) Å	µ = 0.27 mm^−^^1^
*c* = 17.1669 (4) Å	*T* = 100 K
β = 118.195 (1)°	Block, colourless
*V* = 1397.52 (5) Å^3^	0.36 × 0.19 × 0.16 mm
*Z* = 4	

### Data collection

**Table:**

Bruker SMART APEXII CCD area-detector diffractometer	4303 independent reflections
Radiation source: fine-focus sealed tube	3494 reflections with *I* > 2σ(*I*)
Graphite monochromator	*R*_int_ = 0.047
φ and ω scans	θ_max_ = 30.6°, θ_min_ = 2.4°
Absorption correction: multi-scan (*SADABS*; Bruker, 2009)	*h* = −24→24
*T*_min_ = 0.911, *T*_max_ = 0.959	*k* = −7→7
26795 measured reflections	*l* = −24→24

### Refinement

**Table:**

Refinement on *F*^2^	Primary atom site location: structure-invariant direct methods
Least-squares matrix: full	Secondary atom site location: difference Fourier map
*R*[*F*^2^ > 2σ(*F*^2^)] = 0.056	Hydrogen site location: inferred from neighbouring sites
*wR*(*F*^2^) = 0.156	H-atom parameters constrained
*S* = 1.04	*w* = 1/[σ^2^(*F*_o_^2^) + (0.0914*P*)^2^ + 0.9651*P*] where *P* = (*F*_o_^2^ + 2*F*_c_^2^)/3
4303 reflections	(Δ/σ)_max_ < 0.001
209 parameters	Δρ_max_ = 1.14 e Å^−^^3^
0 restraints	Δρ_min_ = −0.72 e Å^−^^3^

### Special details

**Table:**

Experimental. The crystal was placed in the cold stream of an Oxford Cryosystems Cobra open-flow nitrogen cryostat (Cosier & Glazer, 1986) operating at 100.0 (1) K.
Geometry. All s.u.'s (except the s.u. in the dihedral angle between two l.s. planes) are estimated using the full covariance matrix. The cell s.u.'s are taken into account individually in the estimation of s.u.'s in distances, angles and torsion angles; correlations between s.u.'s in cell parameters are only used when they are defined by crystal symmetry. An approximate (isotropic) treatment of cell s.u.'s is used for estimating s.u.'s involving l.s. planes.
Refinement. Refinement of F^2^ against ALL reflections. The weighted R-factor wR and goodness of fit S are based on F^2^, conventional R-factors R are based on F, with F set to zero for negative F^2^. The threshold expression of F^2^ > 2σ(F^2^) is used only for calculating R-factors(gt) etc. and is not relevant to the choice of reflections for refinement. R-factors based on F^2^ are statistically about twice as large as those based on F, and R- factors based on ALL data will be even larger.

### Fractional atomic coordinates and isotropic or equivalent isotropic displacement parameters (Å^2^)

**Table:**

	*x*	*y*	*z*	*U*_iso_*/*U*_eq_	
S1	0.05985 (3)	0.71221 (8)	0.40375 (3)	0.01356 (13)	
F1	0.39345 (8)	0.2691 (3)	0.47175 (9)	0.0276 (3)	
O1	0.00823 (8)	0.4700 (3)	0.40935 (9)	0.0161 (3)	
O2	−0.26376 (8)	0.8084 (3)	0.36579 (9)	0.0161 (3)	
O3	0.01710 (9)	0.8017 (3)	0.31455 (9)	0.0187 (3)	
O4	0.07250 (9)	0.8822 (3)	0.47276 (9)	0.0207 (3)	
O5	−0.38605 (9)	0.9692 (3)	0.35461 (9)	0.0222 (3)	
C1	0.15987 (12)	0.3544 (4)	0.38064 (12)	0.0163 (4)	
H1A	0.1064	0.2816	0.3380	0.020*	
C2	0.23997 (13)	0.2524 (4)	0.39694 (13)	0.0182 (4)	
H2A	0.2424	0.1061	0.3667	0.022*	
C3	0.31602 (12)	0.3684 (4)	0.45807 (13)	0.0187 (4)	
C4	0.31704 (12)	0.5773 (4)	0.50573 (13)	0.0188 (4)	
H4A	0.3708	0.6504	0.5476	0.023*	
C5	0.23669 (12)	0.6784 (4)	0.49075 (12)	0.0163 (4)	
H5A	0.2346	0.8214	0.5227	0.020*	
C6	0.15971 (11)	0.5657 (3)	0.42815 (12)	0.0140 (3)	
C7	−0.08408 (11)	0.4728 (3)	0.36472 (12)	0.0139 (3)	
C8	−0.12939 (11)	0.6500 (4)	0.38628 (12)	0.0147 (3)	
H8A	−0.0998	0.7771	0.4288	0.018*	
C9	−0.22037 (11)	0.6323 (3)	0.34251 (12)	0.0139 (3)	
C10	−0.35402 (12)	0.8098 (4)	0.32803 (13)	0.0169 (4)	
C11	−0.40187 (12)	0.6269 (4)	0.25971 (12)	0.0177 (4)	
H11A	−0.4642	0.6291	0.2315	0.021*	
C12	−0.36115 (12)	0.4529 (4)	0.23449 (12)	0.0156 (3)	
C13	−0.26579 (12)	0.4483 (4)	0.27933 (12)	0.0146 (3)	
C14	−0.21569 (12)	0.2714 (4)	0.26141 (12)	0.0160 (4)	
H14A	−0.2447	0.1423	0.2196	0.019*	
C15	−0.12520 (12)	0.2829 (4)	0.30367 (12)	0.0157 (3)	
H15A	−0.0918	0.1634	0.2912	0.019*	
C16	−0.41273 (13)	0.2751 (4)	0.16052 (14)	0.0209 (4)	
H16A	−0.4755	0.2979	0.1408	0.031*	
H16B	−0.3959	0.1027	0.1811	0.031*	
H16C	−0.4006	0.3084	0.1112	0.031*	

### Atomic displacement parameters (Å^2^)

**Table:**

	*U*^11^	*U*^22^	*U*^33^	*U*^12^	*U*^13^	*U*^23^
S1	0.0139 (2)	0.0141 (2)	0.0135 (2)	0.00107 (14)	0.00718 (18)	0.00011 (15)
F1	0.0170 (6)	0.0382 (8)	0.0280 (7)	0.0093 (5)	0.0110 (5)	0.0014 (6)
O1	0.0137 (6)	0.0158 (6)	0.0191 (7)	0.0007 (5)	0.0079 (5)	0.0029 (5)
O2	0.0129 (6)	0.0189 (7)	0.0170 (6)	0.0011 (5)	0.0075 (5)	−0.0029 (5)
O3	0.0185 (7)	0.0217 (7)	0.0162 (7)	0.0037 (5)	0.0085 (6)	0.0058 (5)
O4	0.0216 (7)	0.0206 (7)	0.0213 (7)	0.0000 (5)	0.0111 (6)	−0.0068 (6)
O5	0.0172 (6)	0.0259 (8)	0.0236 (7)	0.0024 (5)	0.0098 (6)	−0.0048 (6)
C1	0.0182 (9)	0.0188 (9)	0.0127 (8)	0.0001 (7)	0.0079 (7)	−0.0002 (7)
C2	0.0211 (9)	0.0192 (9)	0.0170 (9)	0.0033 (7)	0.0112 (8)	0.0010 (7)
C3	0.0140 (8)	0.0264 (10)	0.0171 (9)	0.0053 (7)	0.0085 (7)	0.0041 (8)
C4	0.0139 (8)	0.0240 (10)	0.0172 (9)	−0.0004 (7)	0.0063 (7)	0.0011 (7)
C5	0.0167 (8)	0.0182 (9)	0.0141 (8)	−0.0005 (6)	0.0074 (7)	0.0002 (7)
C6	0.0134 (8)	0.0170 (8)	0.0119 (8)	0.0012 (6)	0.0063 (7)	0.0005 (6)
C7	0.0135 (8)	0.0158 (8)	0.0126 (8)	0.0012 (6)	0.0064 (7)	0.0032 (6)
C8	0.0154 (8)	0.0165 (8)	0.0131 (8)	−0.0004 (6)	0.0073 (7)	−0.0007 (7)
C9	0.0147 (8)	0.0152 (8)	0.0134 (8)	0.0006 (6)	0.0080 (7)	0.0006 (6)
C10	0.0142 (8)	0.0213 (9)	0.0161 (9)	0.0014 (6)	0.0079 (7)	0.0011 (7)
C11	0.0130 (8)	0.0225 (9)	0.0162 (9)	−0.0020 (7)	0.0059 (7)	−0.0015 (7)
C12	0.0163 (8)	0.0192 (9)	0.0118 (8)	−0.0023 (6)	0.0069 (7)	−0.0003 (7)
C13	0.0168 (8)	0.0161 (8)	0.0123 (8)	−0.0004 (6)	0.0079 (7)	0.0005 (6)
C14	0.0196 (9)	0.0157 (8)	0.0134 (8)	−0.0007 (6)	0.0084 (7)	−0.0014 (6)
C15	0.0201 (9)	0.0152 (8)	0.0141 (8)	0.0011 (6)	0.0101 (7)	−0.0004 (6)
C16	0.0186 (9)	0.0232 (10)	0.0189 (9)	−0.0044 (7)	0.0071 (8)	−0.0046 (7)

### Geometric parameters (Å, º)

**Table:**

S1—O4	1.4249 (14)	C5—H5A	0.9500
S1—O3	1.4318 (14)	C7—C8	1.386 (2)
S1—O1	1.6003 (14)	C7—C15	1.389 (3)
S1—C6	1.7572 (18)	C8—C9	1.390 (2)
F1—C3	1.354 (2)	C8—H8A	0.9500
O1—C7	1.407 (2)	C9—C13	1.398 (3)
O2—C9	1.375 (2)	C10—C11	1.448 (3)
O2—C10	1.379 (2)	C11—C12	1.355 (3)
O5—C10	1.216 (2)	C11—H11A	0.9500
C1—C2	1.389 (3)	C12—C13	1.454 (2)
C1—C6	1.393 (3)	C12—C16	1.496 (3)
C1—H1A	0.9500	C13—C14	1.411 (2)
C2—C3	1.382 (3)	C14—C15	1.381 (3)
C2—H2A	0.9500	C14—H14A	0.9500
C3—C4	1.379 (3)	C15—H15A	0.9500
C4—C5	1.397 (3)	C16—H16A	0.9800
C4—H4A	0.9500	C16—H16B	0.9800
C5—C6	1.392 (3)	C16—H16C	0.9800
			
O4—S1—O3	118.38 (9)	C7—C8—H8A	121.7
O4—S1—O1	109.63 (8)	C9—C8—H8A	121.7
O3—S1—O1	108.24 (8)	O2—C9—C8	115.48 (16)
O4—S1—C6	109.75 (9)	O2—C9—C13	121.48 (15)
O3—S1—C6	110.88 (8)	C8—C9—C13	123.03 (16)
O1—S1—C6	98.01 (8)	O5—C10—O2	116.29 (17)
C7—O1—S1	118.71 (11)	O5—C10—C11	126.04 (17)
C9—O2—C10	121.29 (15)	O2—C10—C11	117.66 (16)
C2—C1—C6	118.51 (18)	C12—C11—C10	122.46 (17)
C2—C1—H1A	120.7	C12—C11—H11A	118.8
C6—C1—H1A	120.7	C10—C11—H11A	118.8
C3—C2—C1	118.60 (18)	C11—C12—C13	118.27 (17)
C3—C2—H2A	120.7	C11—C12—C16	120.97 (17)
C1—C2—H2A	120.7	C13—C12—C16	120.73 (16)
F1—C3—C4	118.67 (18)	C9—C13—C14	117.50 (16)
F1—C3—C2	117.72 (18)	C9—C13—C12	118.70 (16)
C4—C3—C2	123.61 (17)	C14—C13—C12	123.79 (17)
C3—C4—C5	118.10 (18)	C15—C14—C13	121.10 (17)
C3—C4—H4A	121.0	C15—C14—H14A	119.4
C5—C4—H4A	120.9	C13—C14—H14A	119.4
C6—C5—C4	118.71 (18)	C14—C15—C7	118.56 (16)
C6—C5—H5A	120.6	C14—C15—H15A	120.7
C4—C5—H5A	120.6	C7—C15—H15A	120.7
C5—C6—C1	122.44 (16)	C12—C16—H16A	109.5
C5—C6—S1	117.74 (14)	C12—C16—H16B	109.5
C1—C6—S1	119.65 (14)	H16A—C16—H16B	109.5
C8—C7—C15	123.20 (16)	C12—C16—H16C	109.5
C8—C7—O1	120.08 (16)	H16A—C16—H16C	109.5
C15—C7—O1	116.60 (15)	H16B—C16—H16C	109.5
C7—C8—C9	116.60 (17)		
			
O4—S1—O1—C7	88.16 (14)	C10—O2—C9—C8	−179.26 (16)
O3—S1—O1—C7	−42.30 (14)	C10—O2—C9—C13	0.4 (3)
C6—S1—O1—C7	−157.48 (13)	C7—C8—C9—O2	179.28 (15)
C6—C1—C2—C3	−1.5 (3)	C7—C8—C9—C13	−0.3 (3)
C1—C2—C3—F1	−178.69 (17)	C9—O2—C10—O5	178.24 (17)
C1—C2—C3—C4	1.7 (3)	C9—O2—C10—C11	−3.0 (2)
F1—C3—C4—C5	179.75 (17)	O5—C10—C11—C12	−179.18 (19)
C2—C3—C4—C5	−0.6 (3)	O2—C10—C11—C12	2.2 (3)
C3—C4—C5—C6	−0.6 (3)	C10—C11—C12—C13	1.2 (3)
C4—C5—C6—C1	0.7 (3)	C10—C11—C12—C16	−176.90 (18)
C4—C5—C6—S1	−174.62 (14)	O2—C9—C13—C14	−178.34 (16)
C2—C1—C6—C5	0.4 (3)	C8—C9—C13—C14	1.3 (3)
C2—C1—C6—S1	175.59 (14)	O2—C9—C13—C12	3.1 (3)
O4—S1—C6—C5	−18.55 (17)	C8—C9—C13—C12	−177.28 (17)
O3—S1—C6—C5	114.09 (15)	C11—C12—C13—C9	−3.8 (3)
O1—S1—C6—C5	−132.82 (15)	C16—C12—C13—C9	174.28 (17)
O4—S1—C6—C1	166.00 (14)	C11—C12—C13—C14	177.74 (18)
O3—S1—C6—C1	−61.35 (17)	C16—C12—C13—C14	−4.2 (3)
O1—S1—C6—C1	51.73 (16)	C9—C13—C14—C15	−1.1 (3)
S1—O1—C7—C8	−60.4 (2)	C12—C13—C14—C15	177.33 (17)
S1—O1—C7—C15	123.36 (15)	C13—C14—C15—C7	0.1 (3)
C15—C7—C8—C9	−0.8 (3)	C8—C7—C15—C14	0.9 (3)
O1—C7—C8—C9	−176.72 (15)	O1—C7—C15—C14	176.96 (16)

### Hydrogen-bond geometry (Å, º)

**Table:**

*D*—H···*A*	*D*—H	H···*A*	*D*···*A*	*D*—H···*A*
C1—H1*A*···O3^i^	0.95	2.48	3.314 (2)	147
C4—H4*A*···O5^ii^	0.95	2.57	3.214 (3)	126
C8—H8*A*···O4^ii^	0.95	2.37	3.288 (3)	162
C11—H11*A*···O5^iii^	0.95	2.45	3.349 (3)	158
C15—H15*A*···O3^iv^	0.95	2.59	3.502 (3)	160
C16—H16*A*···O5^iii^	0.98	2.60	3.522 (3)	157

Symmetry codes: (i) −*x*, *y*−1/2, −*z*+1/2; (ii) −*x*, −*y*+2, −*z*+1; (iii) −*x*−1, *y*−1/2, −*z*+1/2; (iv) *x*, *y*−1, *z*.
